# The role of enteral nutrition and parenteral nutrition in the management of inflammatory bowel disease: a bibliometric analysis (1999–2025)

**DOI:** 10.3389/fnut.2026.1767514

**Published:** 2026-03-06

**Authors:** Lu Zhong, Yingjie Qing, Jie Liu

**Affiliations:** 1 State Key Laboratory of Technologies for Chinese Medicine Pharmaceutical Process Control and Intelligent Manufacture, Nanjing University of Chinese Medicine, Nanjing, China; 2 School of Pharmacy, Nanjing University of Chinese Medicine, Nanjing, China; 3 Department of Pharmacy, Nanjing Pukou People's Hospital, Liangjiang Hospital, Southeast University, Nanjing, China

**Keywords:** bibliometric analysis, enteral nutrition, inflammatory bowel disease, parenteral nutrition, visualization

## Abstract

**Objectives:**

As a chronic disease often accompanied by malnutrition, Inflammatory Bowel Disease (IBD) has encountered many difficulties in long-term management. Enteral nutrition (EN) and parenteral nutrition (PN) are important methods to help improve nutritional status and support the remission of the disease. However, although increasing attention has been directed toward this kind of nutritional therapy, there is still a lack of detailed and accurate bibliometric analysis in this field. Therefore, the goal of this study is to find out the research trends and hot spots, and provide reference for future academic exploration and clinical practice.

**Methods:**

On November 26th, 2025, publications concerning EN, PN in IBD published from 1999 to 2025 were retrieved from two databases: Web of Science Core Collection and Scopus. In this study, R software, Microsoft Excel, VOSviewer and CiteSpace are used to measure and visually analyze the articles.

**Results:**

A total of 3,245 documents were reviewed, indicating a trend of power-law growth. The USA and UK lead in global collaboration, with China being third in volume but with less cooperative intensity. The study identifies a clear progression from early “total parenteral nutrition” and “corticosteroids” to “exclusive enteral nutrition”. Citation bursts occurring in the years 2020 to 2025 included “Mediterranean diet,” “gut microbiome,” and “Crohn’s disease exclusion diet,” pointing to an emerging focus in personalized, whole-food-based therapies and microbiome modification.

**Conclusion:**

The research on nutritional therapy of IBD demonstrates a rapid increase, which shows that it is becoming more and more important in helping patients control their illness. In the future, we need more high-quality research to turn these data into useful and multidisciplinary treatment guidelines.

## 1 Introduction

Inflammatory bowel disease (IBD), mainly including Crohn’s disease (CD) and ulcerative colitis is a chronic and recurrent systemic disease, characterized by uncontrollable inflammation in gastrointestinal tract (1, 2). Malnutrition is a common and serious problem in patients with IBD because the digestive system is affected, and inflammation significantly increases energy expenditure, which has a great impact on their recovery, quality of life and treatment effect (3). Therefore, nutritional intervention has become an important part of multidisciplinary treatment of IBD (4, 5). Enteral nutrition (EN) and parenteral nutrition (PN) are not only supportive measures to improve malnutrition, but also important treatment methods, which can help relieve the disease and promote the healing of intestinal mucosa (6, 7). Previously, total parenteral nutrition (TPN) was often used to “rest” the intestine (8). However, accumulating evidence indicates that EN is a superior alternative. Exclusive enteral nutrition (EEN), in particular, has become the first-line therapy for children’s CD (9). It is safe and can help the intestinal mucosa heal (10). Recently, new dietary interventions, such as the Crohn’s disease exclusion diet (CDED), and research on intestinal flora have made nutritional therapy more possible (11, 12). Nutritional therapy is no longer simply to provide calories, but to develop in the direction of precise nutrition and regulation of flora (13). In the past two decades, academic research in this field has garnered increasing attention. The number of papers published every year is increasing, which shows that knowledge is demonstrating a rapid increase. But so much information also poses a significant challenge for clinicians and researchers, it is difficult for them to grasp the whole development of the whole field at once. Although the traditional systematic review can summarize specific clinical problems in detail, it often fails to grasp the general direction of the overall structure, dynamic changes and cooperation network of the discipline. At present, there is still a lack of comprehensive bibliometric analysis on the role of EN and PN in the treatment of IBD. Therefore, this study plans to comprehensively evaluate the global research situation from 1999 to 2025 with the tools of VOSviewer and CiteSpace. By visualizing the knowledge structure, finding out influential research and discovering new hot spots, such as personalized diet strategy and intestinal flora interaction, we hope to provide useful information to help future academic research and optimize clinical decision-making (14). It is important to note that while this bibliometric analysis maps the landscape of research trends and academic attention, it is inherently descriptive and reflects scientific impact (citations) rather than directly evaluating clinical efficacy or patient outcomes.

## 2 Materials and methods

### 2.1 Data source

The information we used in our research was found from the Web of Science Core Collection (WoSCC) and Scopus. WoSCC is recognized as a very reliable citation database all over the world, which can provide a stable, accurate and comprehensive index to help people do research and exploration better (15). Scopus is also a top-level abstract and citation database, which contains well-organized research contents and rich academic materials (16). These two leading academic databases follow strict content selection and maintenance rules to ensure that the data we use is rigorous and comprehensive.

### 2.2 Search strategy

We used these words when searching: (TS = (“inflammatory bowel disease*” OR “Crohn* disease” OR “ulcerative colitis” OR IBD) AND (“enteral nutrition*” OR “enteral feeding*” OR “tube feeding*” OR “parenteral nutrition*” OR “parenteral feeding*” OR “intravenous feeding*” OR “total parenteral nutrition” OR TPN OR “exclusive enteral nutrition” OR “partial enteral nutrition” OR EEN OR PEN)). The search scope is limited to Articles classified as “article” or “Review” and written in English, which were published from January 1, 1999 to November 26, 2025. After removing duplicate and irrelevant items, we found 1,573 papers from WoSCC and 2,612 papers from Scopus. We obtained 3,245 publications in total through this initial search. Subsequently, we downloaded the results in plain text format. Both search execution and data downloading were in one single day on November 26, 2025.

### 2.3 Data selection and standardization

**Figure 1:**
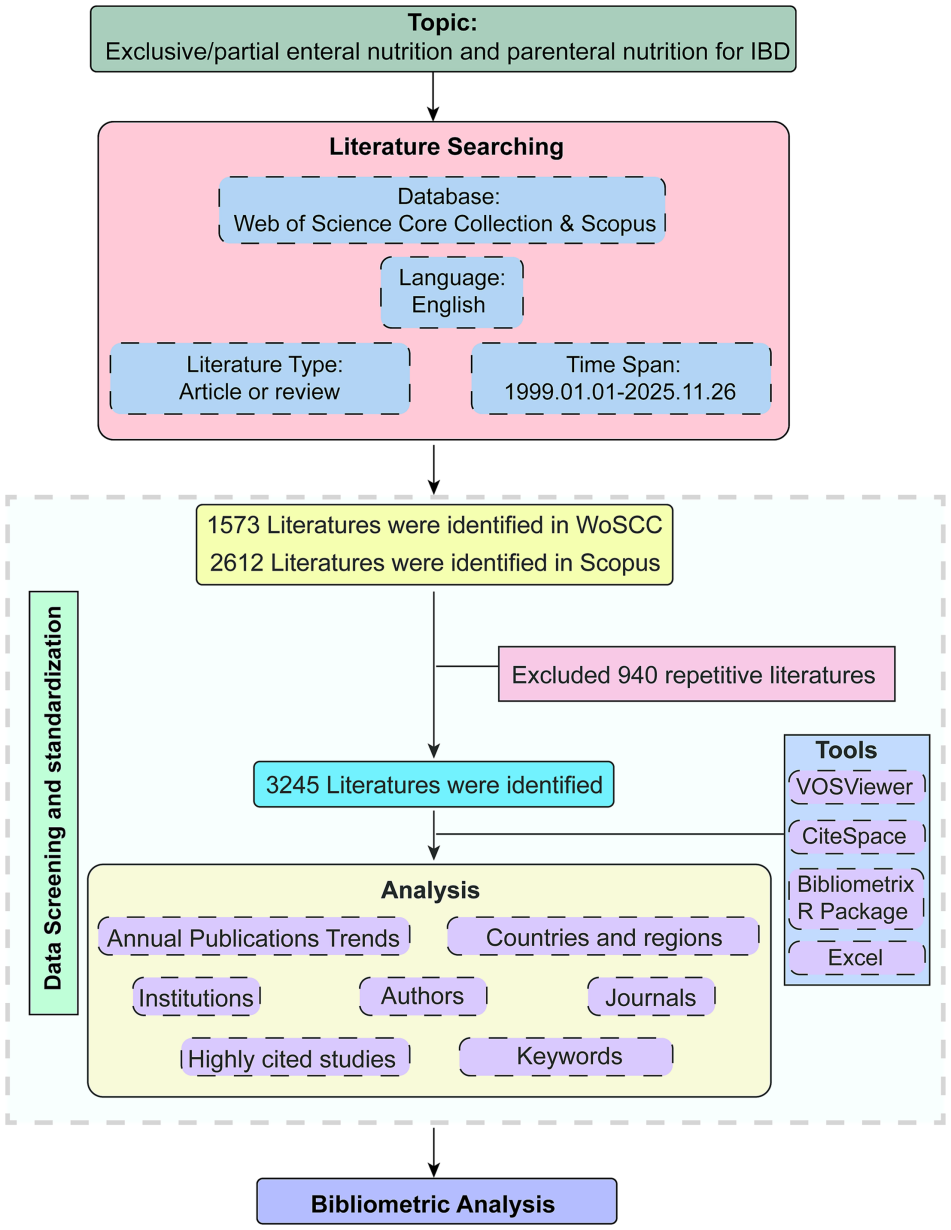
Flow chart of literature search and bibliometric analysis. The selection process details the identification, screening, and inclusion of records from WoSCC and Scopus.

Data selection was strictly governed by inclusion and exclusion criteria applied directly through the advanced search filters of the WoSCC and Scopus databases. The selection rule is: (1) peer-reviewed original research and review articles; (2) the primary research topic or intervention focused specifically on EN (including exclusive and partial enteral nutrition) or PN; and (3) the study population or context was explicitly related to IBD. Conversely, the automated exclusion criteria filtered out non-peer-reviewed document types (e.g., meeting abstracts, editorials) and retracted publications. After retrieving raw data from WoSCC and Scopus, the datasets were merged. Duplicate removal was performed using the bibliometrix R package, identifying 940 duplicates based on title and DOI matching. It is important to note that no subsequent manual, full-text screening was performed. The remaining 3,245 unique records represent the exact and final dataset utilized for all downstream bibliometric analyses (Figure 1).

### 2.4 Data analysis

In this study, we mainly used three softwares: Bibliometrix R Package (Version 4.2.2), VOSviewer (Version 1.6.20) and CiteSpace (Version 6.2. R6), which are responsible for different but extensive analysis tasks. We choose Bibliometrix software, because it is made on the R platform, which can help us to process data quickly, draw scientific charts, calculate important bibliometrics indicators, and generate thematic evolution charts. In addition, we also use Microsoft Excel to make some statistical charts. VOSviewer is a Java-based program, which is used to establish and display the bibliometrics network, such as the cooperation between countries and institutions, the co-citation relationship of journals, and the relationship between keywords (17). CiteSpace was utilized to visualize knowledge domains and identify emerging trends through document co-citation analysis (DCA), cluster analysis, and Kleinberg’s burst detection algorithms (18). Using these three tools together, we can give full play to their respective strengths and combine strict data analysis, intuitive charts and trend prediction. In this way, our research will be more reliable and the field of vision of literature investigation will be broadened. To ensure the reproducibility of our bibliometric mapping, specific software parameters were established and explicitly recorded. In VOSviewer, full counting was utilized to evaluate the absolute weight of co-authored or multi-cited documents. The keyword co-occurrence analysis was based on “All Keywords” (incorporating both Author Keywords and KeyWords Plus) to capture a comprehensive landscape of the research topics, encompassing core therapeutic concepts as well as associated methodological terms. For CiteSpace, the parameters were configured as follows: the time slicing was set from January 1999 to November 26, 2025 with years per slice = 1; the selection criteria utilized the g-index with a scale factor of k = 25; and the pruning method applied was “Pathfinder” to clarify the structural backbone of the networks and reduce excessive visual clutter.

## 3 Results

### 3.1 Publication trends by year

**Figure 2:**
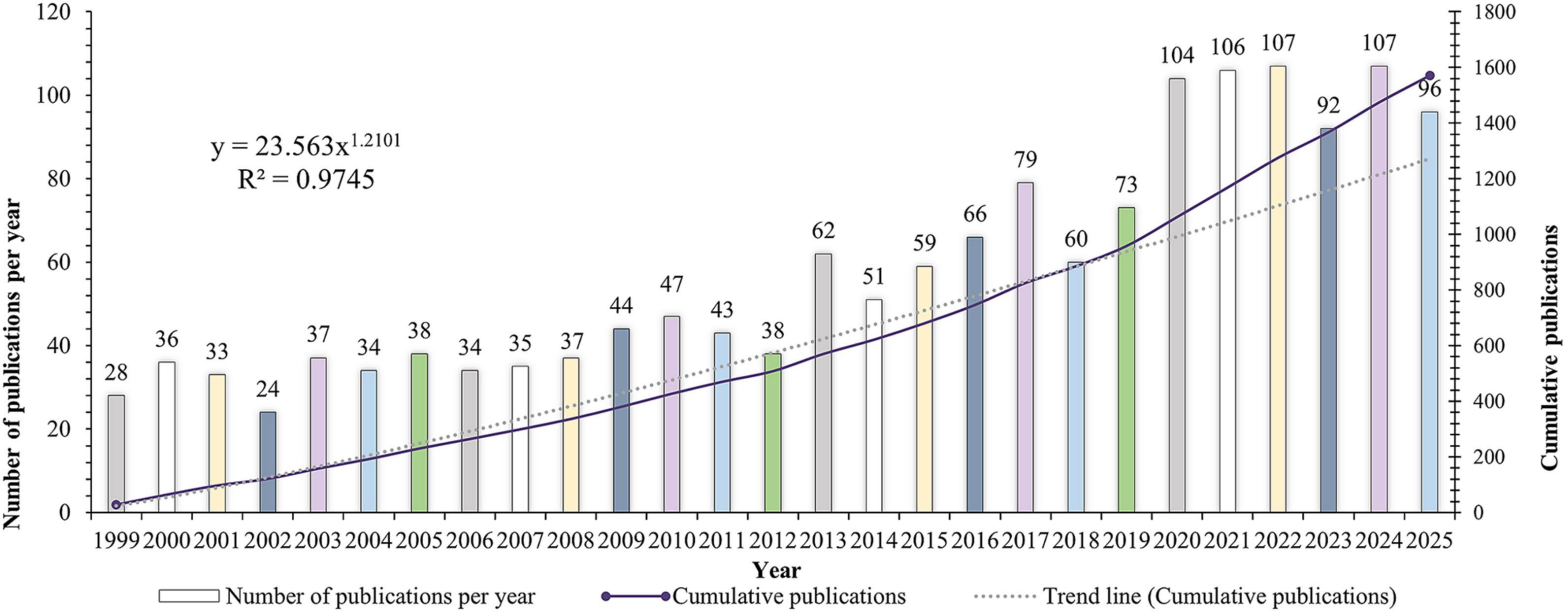
The annual and cumulative number of publications from 1999 to 2025. The bar chart represents the number of publications per year (left *y*-axis), while the line chart represents the cumulative publications (right *y*-axis). The trend line fits a power-law growth model (*y* = 23.563*x*^1.2101^, *R*^2^ = 0.9745). Note: The data for 2025 is partial (retrieved on November 26, 2025) and represents approximately 11 months of publications. Therefore, the apparent count for 2025 does not reflect the full annual volume.

Based on the inclusion/exclusion criteria, 3,245 articles and reviews were ultimately identified from the WoSCC and Scopus (Figure 1). Figure 2 shows the number of papers published on this subject and the overall trend. Before 2013, the number of research in this field was relatively small, and it grew slowly every year, usually no more than 50 articles. However, the steep rise of the cumulative line shows that academic achievements suddenly increased sharply in the next decade, the growth follows a power-law trend (*R*^2^ = 0.9745). By 2020, the number of annual papers exceeded 100 for the first time, reaching 104. Although it declined slightly in 2023 (*n* = 92), it rose to the highest point in history in 2024 (*n* = 107). The number of publications in 2025 reached 96 by November 26, 2025. It is important to note that this represents partial data. Thus, the final annual figure is expected to be higher. The remarkable increase in academic achievements shows that this research field has developed rapidly and attracted increasing attention.

### 3.2 Analysis of national publications

In order to see which countries/regions have contributed the most to this field, we analyzed the publications by different countries/regions and found that the research of 69 countries/regions was included across all publications.

**Figure 3:**
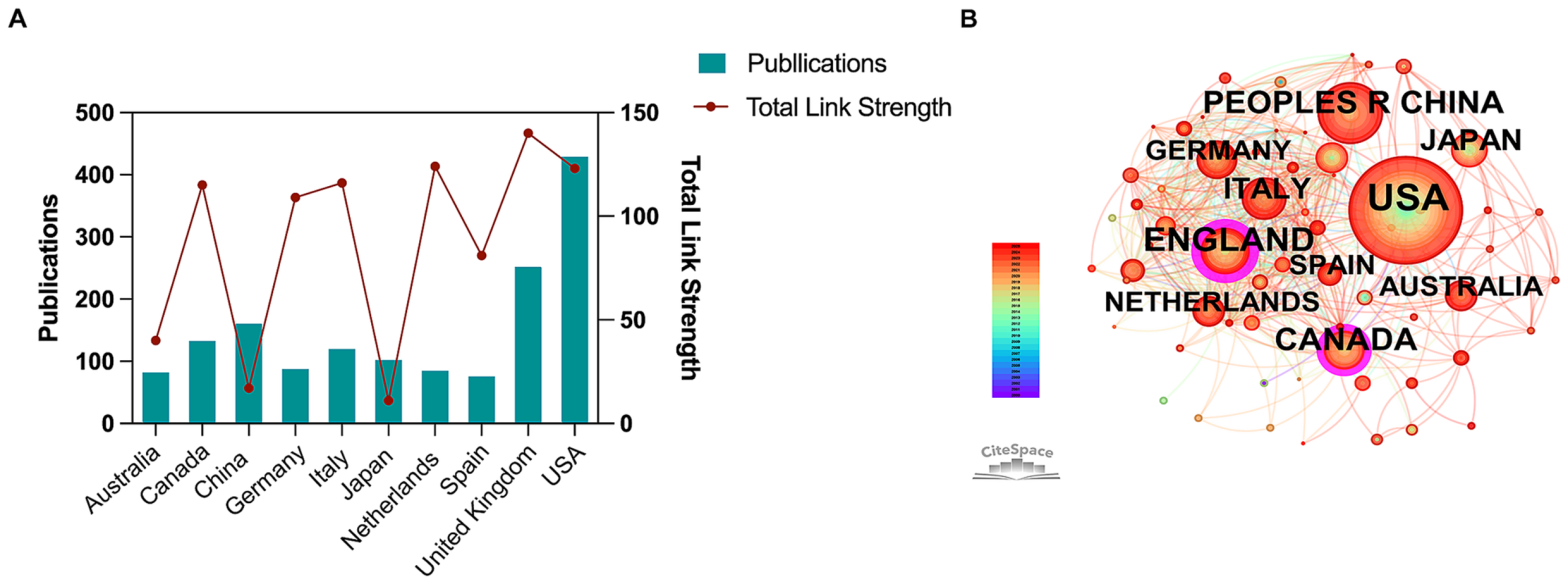
Analysis of countries/regions. **(A)** Top 10 national publications and their Total Link Strength. The blue bars represent publication counts, and the red line represents Total Link Strength. **(B)** Time-overlapping network visualization map of countries/regions. Nodes represent countries; node size is proportional to the number of publications. Lines connecting nodes represent co-authorship links; line thickness indicates the strength of collaboration. Colors indicate the average publication year (blue: earlier; red: more recent). Note: In the WoSCC database, regions such as England, Scotland, Wales, and Northern Ireland are indexed independently, but they are consolidated as the United Kingdom in our geographical discussion.

According to Figure 3A, among the top 10 countries/regions with the most published papers, the USA ranks first with 430 papers, followed by the United Kingdom (253 papers), China (162 papers), Canada (134 papers), Italy (121 papers) and Japan (103 papers). The number of papers in other countries does not exceed 100. We used “Total Link Strength” (TLS)—defined as the cumulative strength of co-authorship links between a specific country and all other countries—to quantify the intensity of international collaboration within the global network. China ranked third in publication volume but recorded a Total Link Strength of 17. In contrast, the UK and USA demonstrated higher link strengths of 140 and 123, respectively. Furthermore, Italy, Canada, Germany, and Netherlands all exhibit a total link strength exceeding 100, indicating their prominent roles in high-intensity international collaboration (Figure 3A).

Furthermore, a timeline-based visualization analysis of country/region-specific publications found that the most contributions in recent years mainly came from North America, Asia and Europe (Figure 3B). Among these continents, in terms of absolute publication volume, the USA, China, and the United Kingdom emerged as the primary contributors in North America, Asia, and Europe, respectively. However, when normalizing for collaboration intensity (TLS per publication), the United Kingdom (0.55) demonstrated a significantly higher degree of international connectivity compared to the USA (0.29) and China (0.10).

### 3.3 Analysis of institution publications

In order to better understand the influence of different institutions on the research of EN, PN and IBD, we sorted out the published data by institutions. Analysis shows that 2037 academic and research institutions around the world have published articles in this field. According to the data in Table 1, Tel Aviv University ranks first with 40 articles published. University of Glasgow and University of Toronto followed closely, with 35 articles each. University of Alberta tied for third place with 30 articles. Nanjing University and University of Washington tied for fourth place, with 26 articles each. However, if the influence is evaluated according to the total link strength, Tel Aviv University, University of Glasgow and University of Alberta rank in the top three, with scores of 75, 53 and 52, respectively.

**Table 1:** The top 10 most productive institutions contributing to research on EN and PN in IBD.

Rank	Institution	Country	Publications	Total link strength
1	Tel Aviv University	Israel	40	75
2	University of Glasgow	United Kingdom	35	53
3	University of Toronto	Canada	35	38
4	University of Alberta	Canada	30	52
5	Nanjing University	China	26	8
6	University of Washington	USA	26	29
7	University of Otago	New Zealand	25	24
8	Children’s Hospital of Philadelphia	USA	24	33
9	University of Pennsylvania	USA	24	33
10	University of Amsterdam	Netherlands	23	42

**Figure 4:**
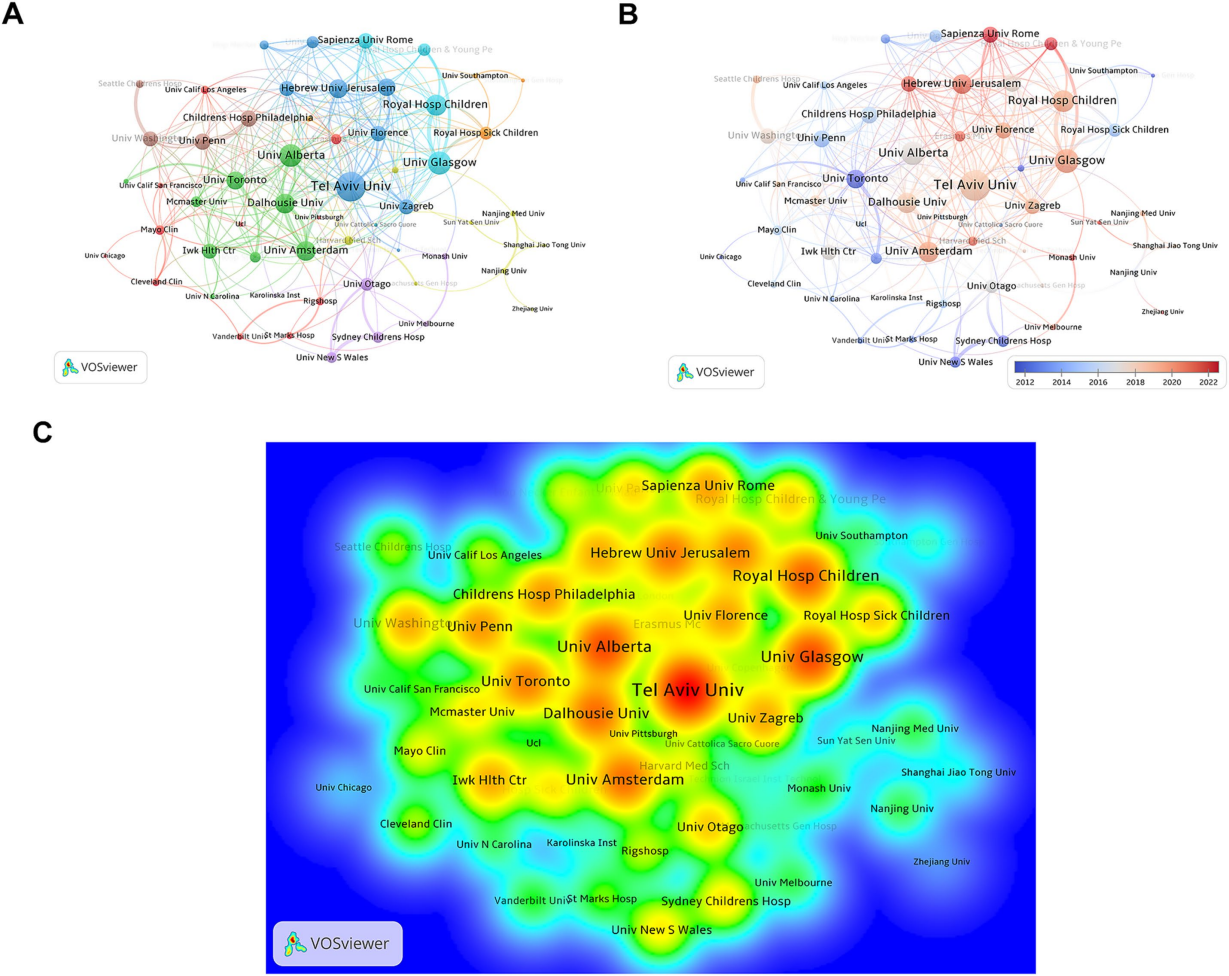
Analysis of institution publications. **(A)** Co-occurrence graph of publishing institutions. Nodes represent institutions, and links represent cooperative relationships. **(B)** Time-overlapping network visualization map of publishing institutions. **(C)** Co-occurrence density view of publishing institutions, where red areas indicate high concentrations of research output and citation impact.

Figure 4 shows the cooperation of research institutions with VOSviewer visual tools. In Figure 4A, the co-occurrence network diagram shows the cooperative relationship between institutions. The size of each circle represents the number of articles published by them, and the connection indicates that there is cooperation between them. Different colors represent different groups of organizations that often work together. It can be seen that they may come from the same area or have academic ties. Figure 4B is an overlay visualization based on average publication year. The color scale in Figure 4B ranges from blue (earlier research around 2012) to red (recent research around 2022). Blue dots represent institutions that have studied in this field for a long time, while red or orange dots represent institutions that have become more and more active in recent years. Figure 4C is a density view, and the “hot spots” areas (red and yellow parts) highlight the institutions that publish the most results. As can be seen from the figure, institutions such as Tel Aviv University, University of Glasgow and University of Alberta are in the central position with high density.

### 3.4 Analysis of journals

**Figure 5:**
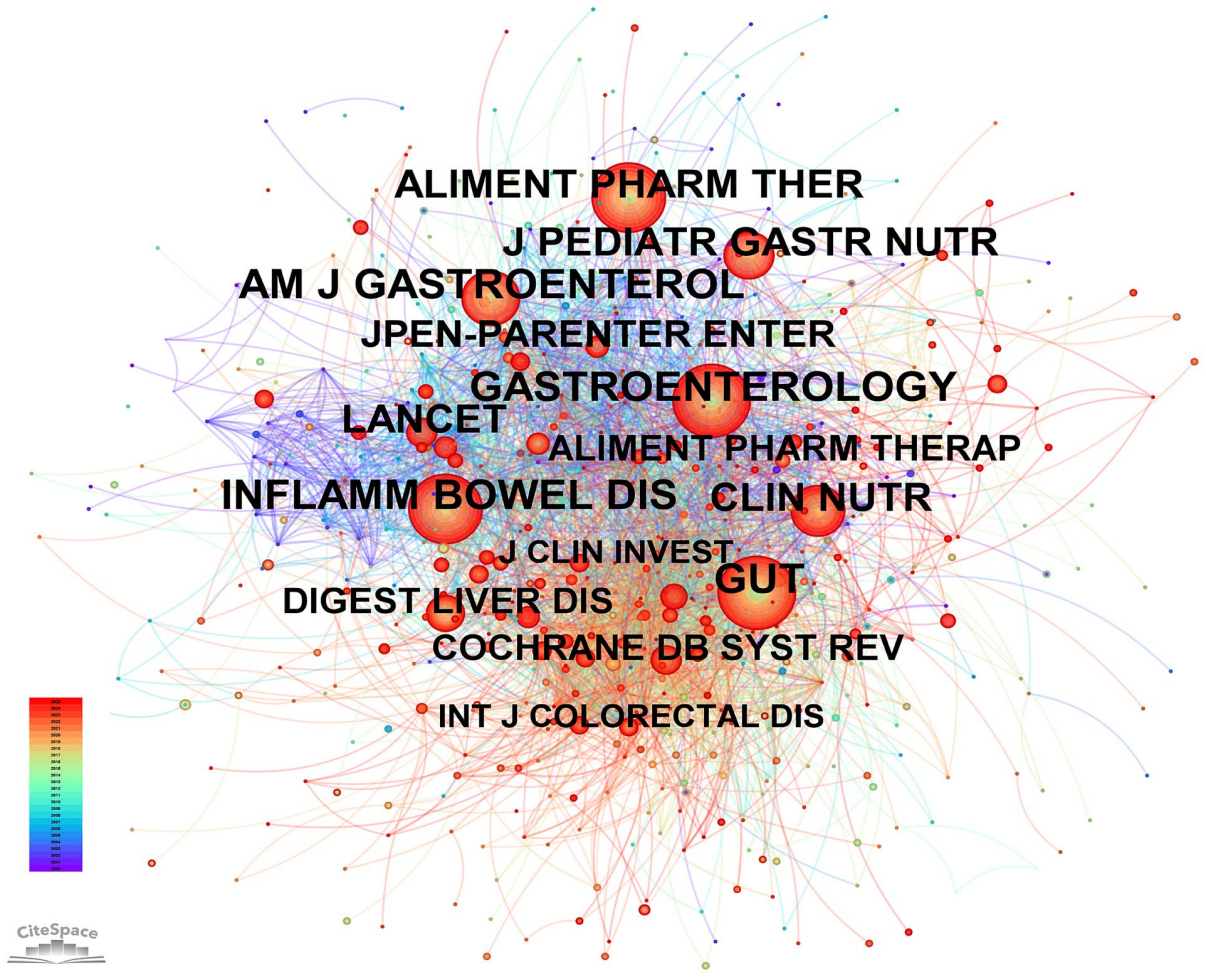
Journal co-citation network visualization (CiteSpace). Nodes represent cited journals, and the node size corresponds to the citation frequency (citation counts). Lines connecting nodes represent co-citation links (journals cited together in the same articles). The colors of the links and nodes rings transition from cool (blue/older years) to warm (red/recent years) tones, illustrating the temporal distribution of citations. Note the prominence of high-impact gastroenterology and nutrition journals forming the backbone of the domain.

Table 2 lists the top 10 most productive journals in the field of enteral and parenteral nutrition in IBD. Nutrients ranked first with 89 articles, followed by Journal of Pediatric Gastroenterology and Nutrition (79 articles) and Inflammatory Bowel Diseases (74 articles). It is worth noting that eight of the top ten journals are classified within the Q1 or Q2 quartiles of the Journal Citation Reports (JCR), which shows that their academic influence and quality are high. Figure 5 shows the journal co-citation network generated by CiteSpace. This analysis reveals the intellectual structure of the field by identifying the most frequently cited journals. In this visualization, node sizes are proportional to citation frequencies. The network reveals extensive citation links among core journals, forming a dense knowledge base. Notably, high-centrality journals such as Alimentary Pharmacology & Therapeutics, Gastroenterology and Gut are in the central position, indicating that they have played a key role in disseminating research findings in this field.

**Table 2:** The top 10 journals with the highest publications on EN and PN in IBD.

Rank	Journals	Publications	JCR
1	Nutrients	89	Q1
2	Journal of Pediatric Gastroenterology and Nutrition	79	Q2
3	Inflammatory Bowel Diseases	74	Q1
4	Clinical Nutrition	44	Q1
5	Journal of Parenteral and Enteral Nutrition	43	Q1
6	Alimentary Pharmacology & Therapeutics	41	Q1
7	Nutrition in Clinical Practice	35	Q3
8	Journal of Crohn’s & Colitis	33	Q1
9	Digestive Diseases and Sciences	31	Q2
10	World Journal of Gastroenterology	25	Q1

### 3.5 Author impact analysis

**Figure 6:**
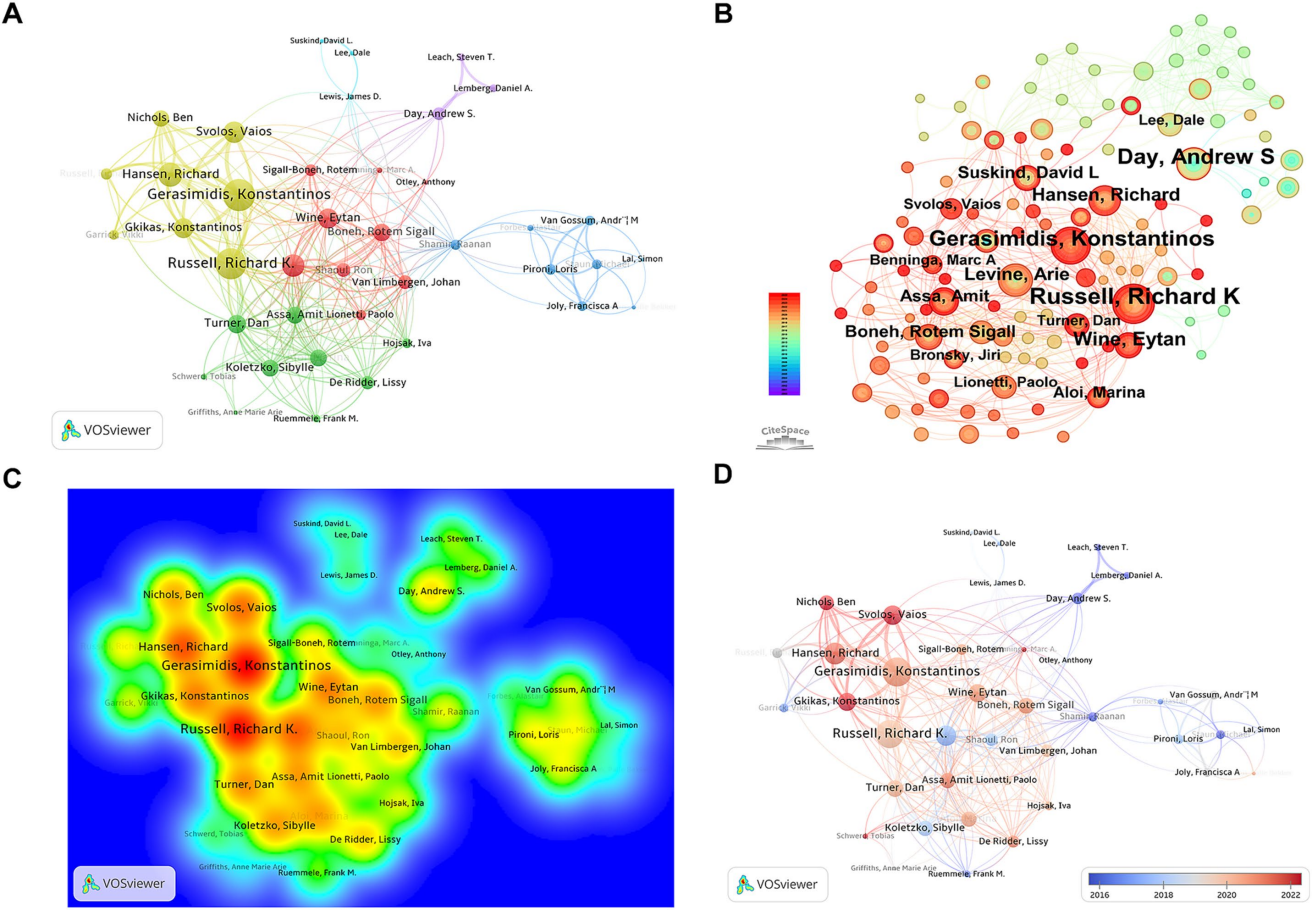
Author impact analysis. **(A)** Network visualization of author collaboration. Node size reflects the number of papers. **(B)** Density visualization identifying top productive authors. **(C)** Author co-occurrence map. **(D)** Time-overlay visualization tracking author activity from 2016 to 2022.

Table 3 presents the top 10 most productive authors in the field of nutritional therapy for IBD. Figure 6 shows the multi-angle visualization of authors’ cooperation in the field of enteral and parenteral nutrition in IBD. Figure 6A is a co-authored network diagram generated by VOSviewer. The circle size represents the amounts of published articles, and the connection line represents the cooperative relationship. Richard K. Russell, Konstantinos Gerasimidis, Eytan Wine and other core authors have a large number of connections as the main nodes, forming an obvious core cooperation group. The density map in Figure 6B further verifies these findings, and the red “hot spots” indicate the most prolific researchers in this field. Figure 6C is generated by CiteSpace, which is mutually verified with the results of VOSviewer, showing a close cooperation network with key opinion leaders such as Andrew S. Day, Richard Hansen as the core. Figure 6D shows the time evolution of research activities, and the color gradually changes from blue (early research) to red (recent research). You can see the authors such as Andrew S. Day, Daniel A. Lemberg laid the foundation, while researchers like Vaios Svolos, Ben Nichols (red nodes) became active contributors recently (about 2021–2022), which promoted the latest progress in this field.

**Table 3:** The top 10 most productive authors on EN and PN in IBD.

Rank	Authors	Publications	Total citations
1	Konstantinos Gerasimidis	42	1,621
2	Richard K. Russell	32	1711
3	Andrew S. Day	31	1,095
4	Weiming Zhu	26	383
5	Richard Hansen	24	568
6	Arie Levine	24	2,132
7	Eytan Wine	24	1736
8	Simon Lal	21	289
9	Jianfeng Gong	20	281
10	Yi Li	19	262

### 3.6 Analysis of keywords and research trends

This part presents a multi-dimensional bibliometric analysis of keywords to visualize the knowledge structure and evolutionary trajectory of enteral and parenteral nutrition in IBD management.

#### 3.6.1 Knowledge structure and clustering

**Figure 7:**
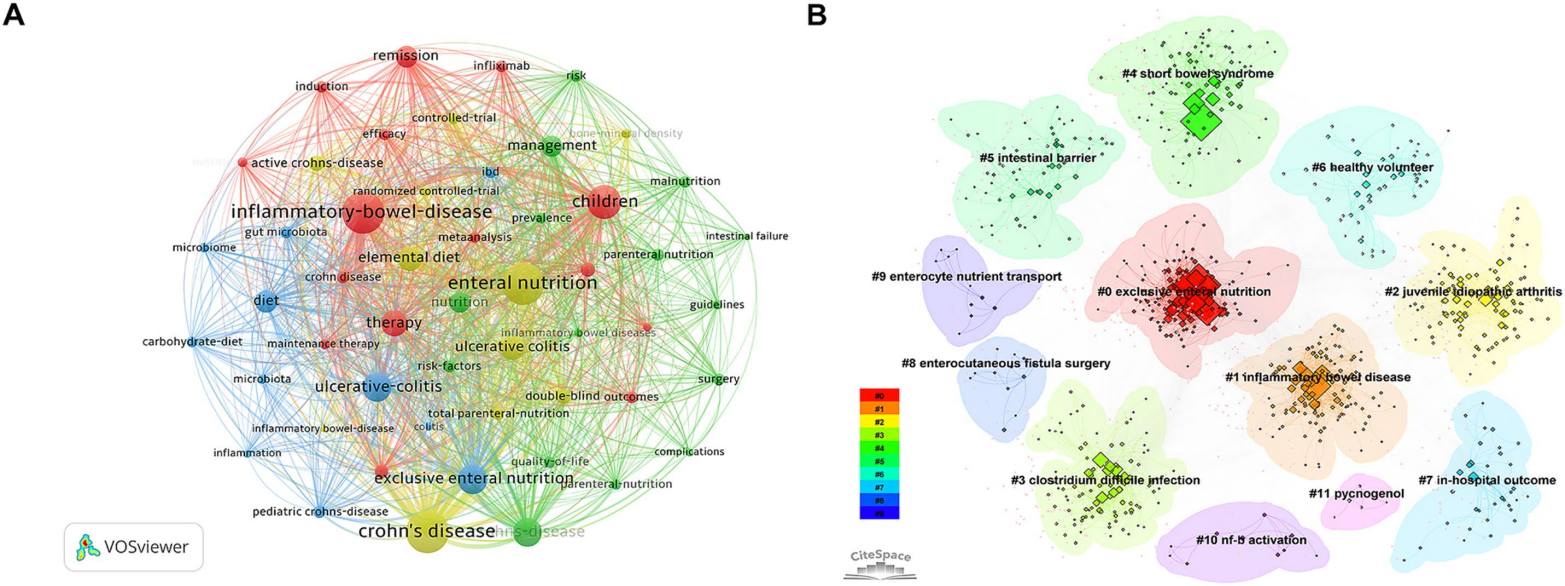
Knowledge structure and clustering. **(A)** Keyword co-occurrence network map generated by VOSviewer, where node size reflects occurrence frequency and lines indicate co-occurrence links. **(B)** Cluster visualization map generated by CiteSpace, categorizing keywords into distinct thematic clusters.

The keyword co-occurrence network delineates the fundamental conceptual framework of the field (Figure 7A). As can be seen, the center of high-frequency nodes included “inflammatory bowel disease”, “enteral nutrition”, “children”, and “remission.” Along with this, the cluster map divides the research area into several thematic clusters (Figure 7B). Cluster #0 “exclusive enteral nutrition” and #1 “inflammatory bowel disease” are by far the largest clusters, followed by other more specific topics such as #4 “short bowel syndrome” and #5 “intestinal barrier,” showing the multiplicity of clinical applications of nutritional support. Cluster #2 (juvenile idiopathic arthritis) reflects shared autoimmune pathways and overlapping dietary therapies in pediatric populations, while cluster #11 (pycnogenol) highlights the exploration of specific antioxidant supplements in IBD management.

#### 3.6.2 Temporal evolution and shifting paradigms

**Figure 8:**
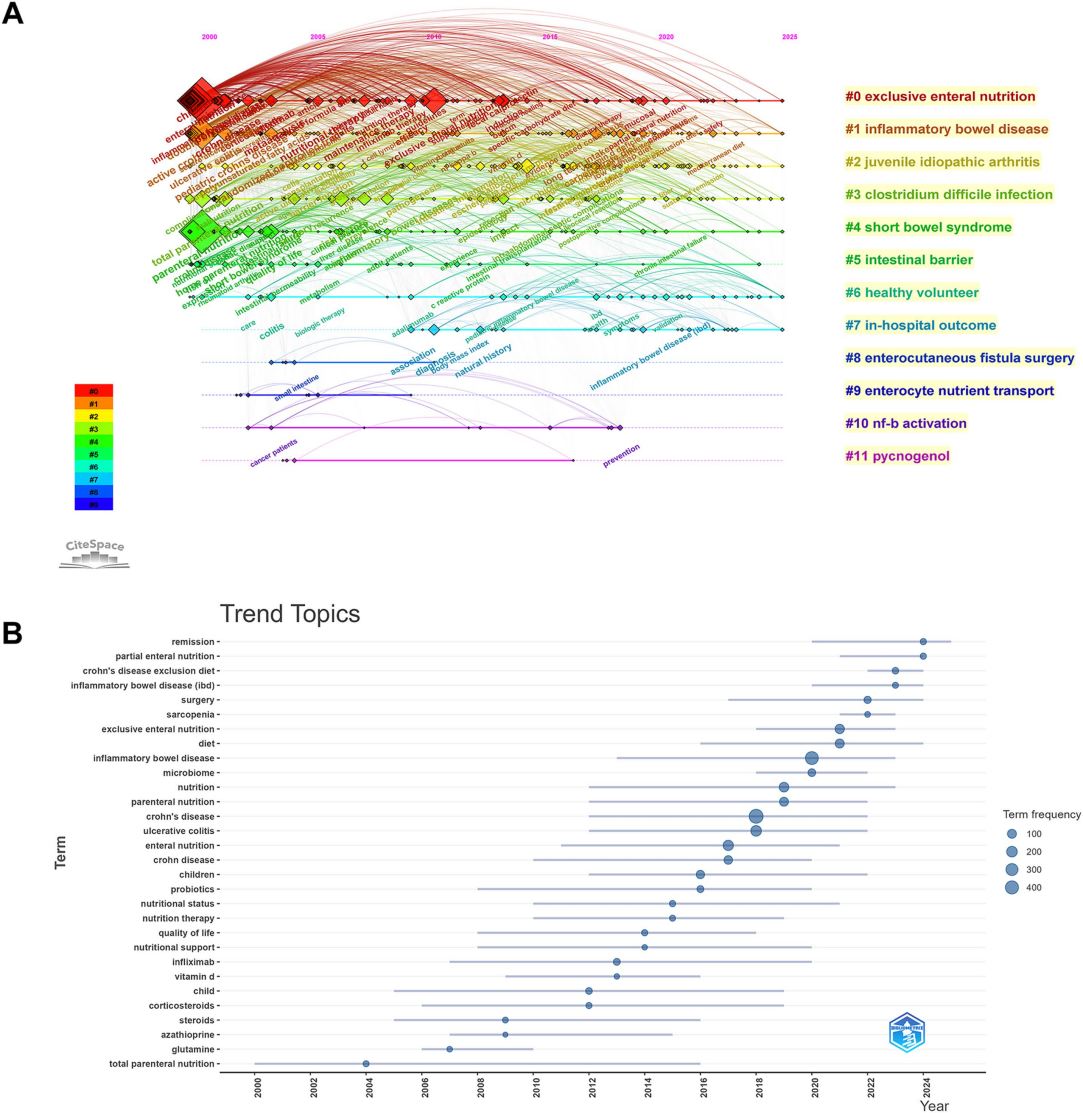
Temporal evolution and shifting paradigms. **(A)** Timeline view of keyword clusters, illustrating the temporal span and evolutionary trajectory of research topics from 2000 to 2025. **(B)** Trend topics analysis showing the lifespan and popularity of high-frequency keywords over time; blue circles represent term frequency, and lines indicate the active timespan of the topic.

The evolution of these themes over time is shown in the timeline view (Figure 8A) as well as in the trend topics analysis (Figure 8B). The timeline indicates that Cluster #0 (exclusive enteral nutrition) continues to be active and represents the core of the research, with a steady stream of publications since 2000 to 2025. The trend topics analysis provides more detail on a major shift in paradigm over the past 20 years. Early studies (2000–2010) were mainly concentrated on “total parenteral nutrition”, “glutamine” and “corticosteroids.” However, following 2015, there is a clear shift in research interest towards “microbiome”, “probiotics” and “quality of life” with the transfusion of concepts from clinical to holistic and microbiome-centric care.

#### 3.6.3 Emerging frontiers and citation bursts

**Figure 9:**
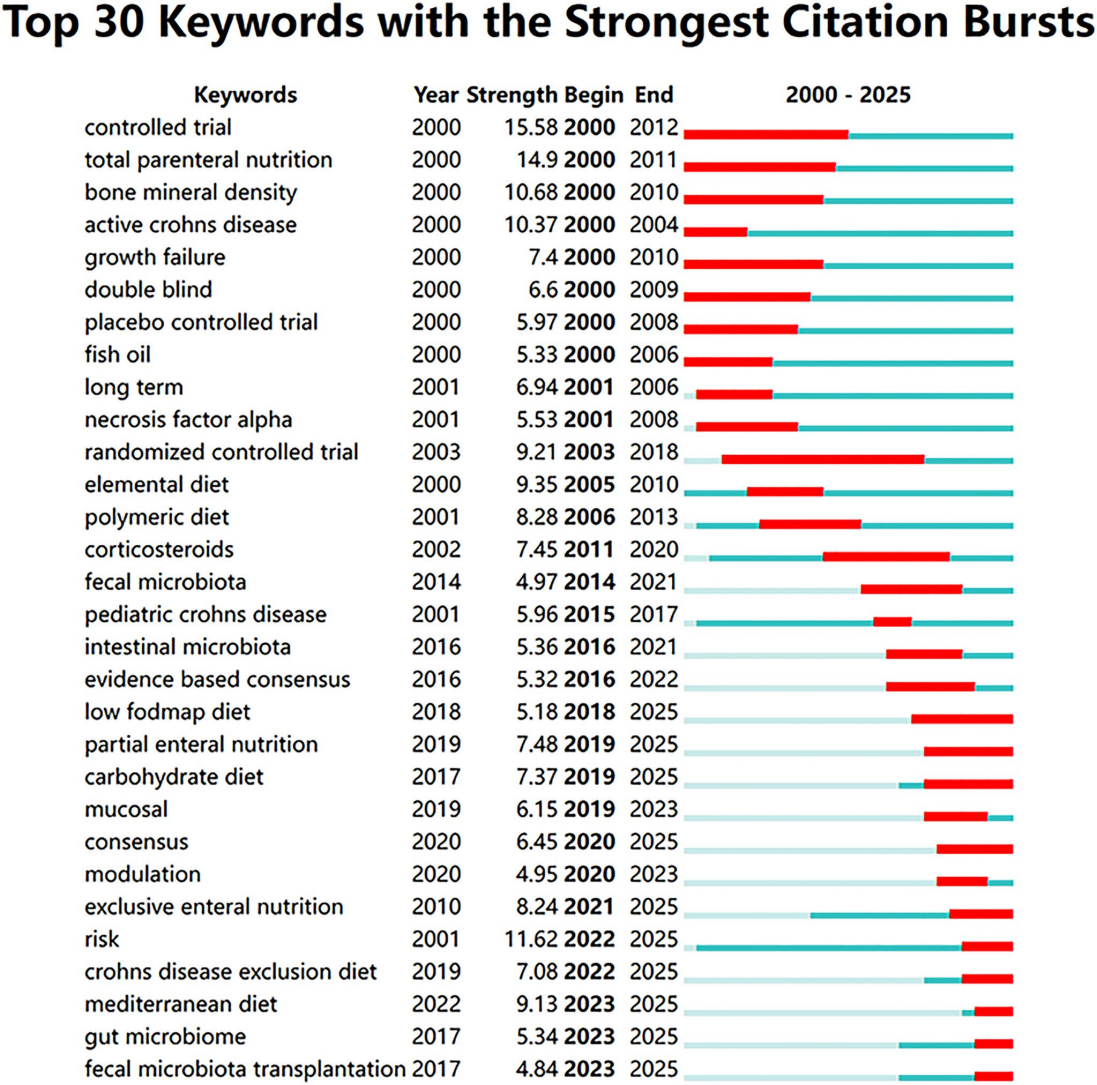
Emerging frontiers and citation bursts. Top 30 keywords with the strongest citation bursts detected by CiteSpace. The blue lines represent the timeline from 2000 to 2025, and the red bars indicate the duration of intense research attention.

Finally, the analysis of citation bursts illuminates the emerging trends and active research frontiers within the field (Figure 9). Terms such as “controlled trial” had strong bursts in the early 2000s, but the latest time slice (2020–2025) shows bursts of the following keywords: “Mediterranean diet” (Strength = 9.13), “gut microbiome”, “fecal microbiota transplantation” and “Crohn’s disease exclusion diet”.

### 3.7 Analysis of co-cited references and intellectual base

**Figure 10:**
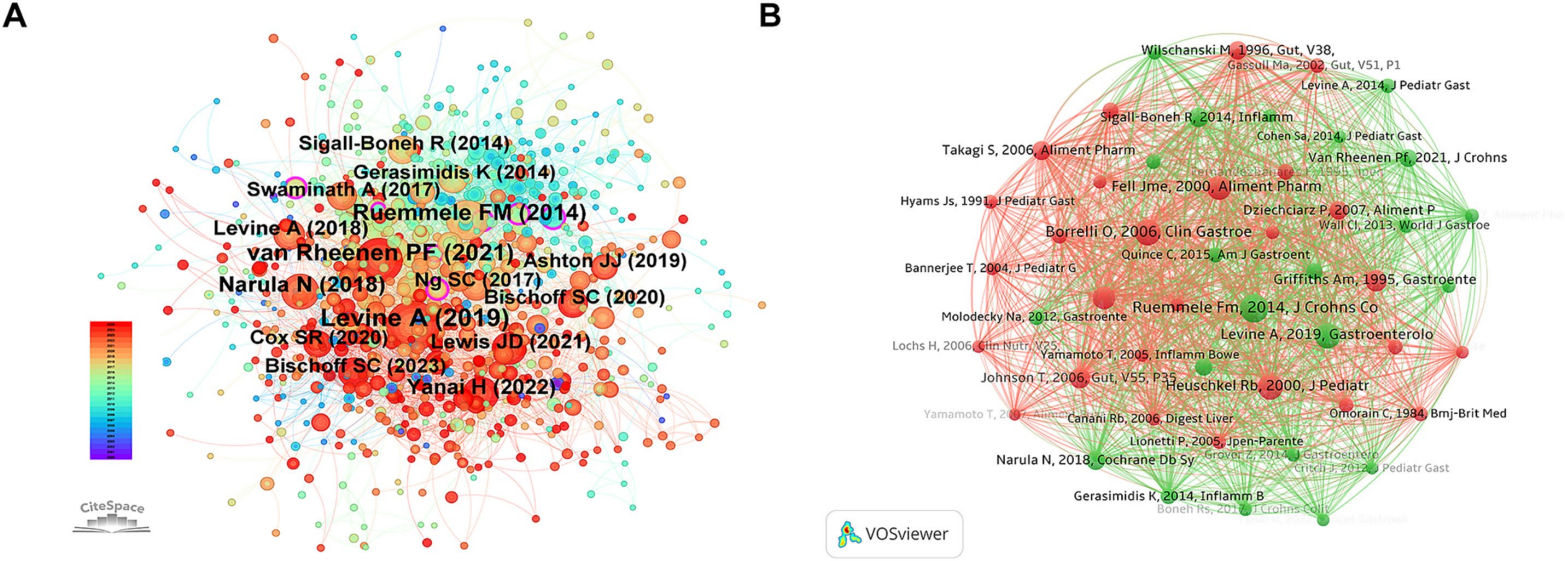
Visualization of the intellectual base and co-cited references. **(A)** Reference co-citation network generated by CiteSpace. The colors of the links correspond to the years of co-citation occurrence, shifting from purple (earlier years) to yellow (recent years). **(B)** Reference co-citation network generated by VOSviewer. Nodes represent cited references, with size proportional to citation frequency.

**Figure 11:**
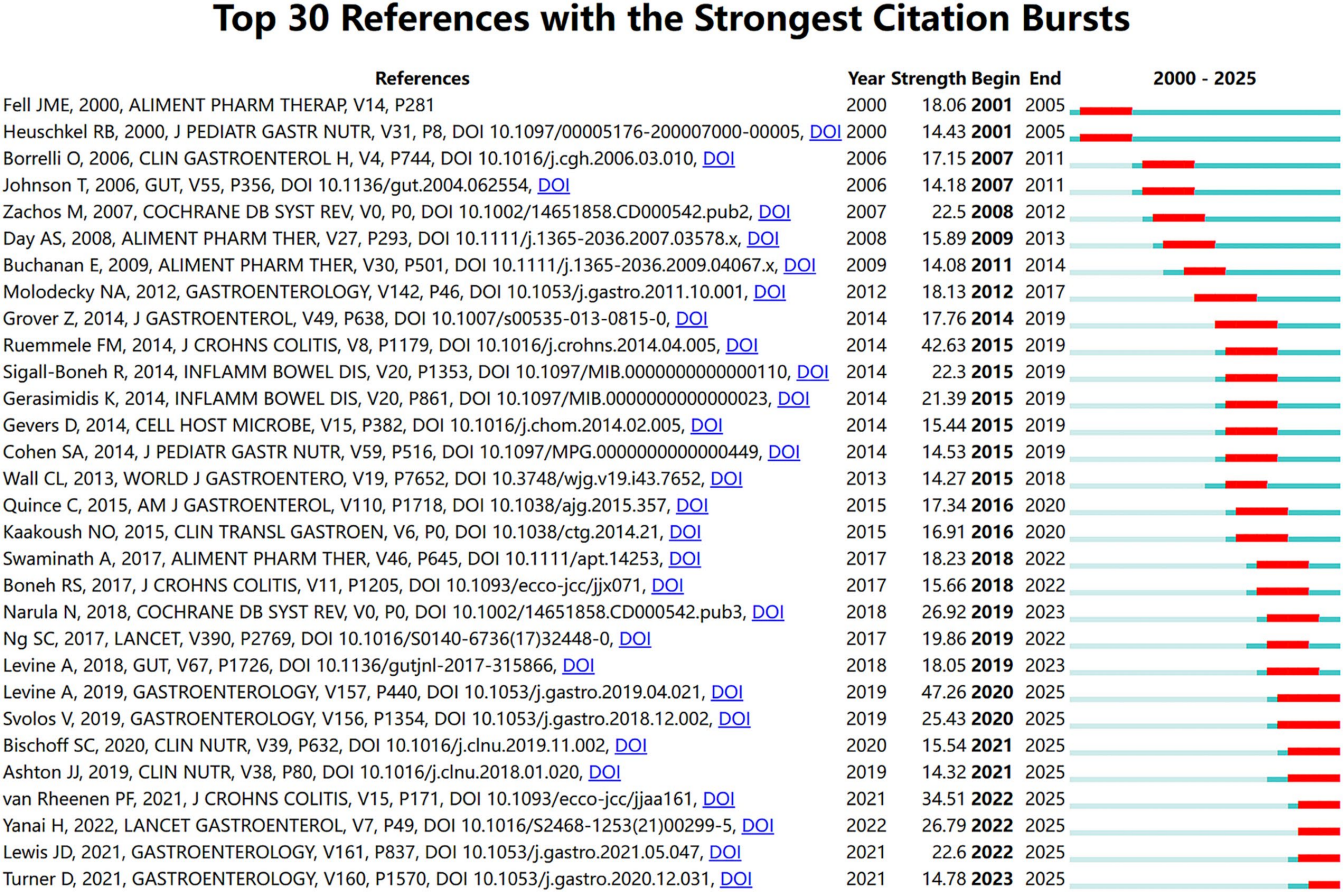
Top 30 references with the strongest citation bursts. The blue lines represent the timeline from 2000 to 2025, with the red part indicating the length of the burst.

Figures 10, 11 show the intellectual base and studies of landmark in the research area of nutritional therapy for IBD with the mashup of reference co-citation analysis.

The reference co-citation in the output of networks generated by CiteSpace and VOSviewer are shown in Figures 10A,B. As is resulted in the network maps, nodes of high centrality are key guidelines and randomized controlled trials (RCTs). The largest nodes are Frank M. Ruemmele (19) and Patrick F. van Rheenen (10) representing the most influential European Crohn’s and Colitis Organization/European Society for Pediatric Gastroenterology, Hepatology and Nutrition (ECCO/ESPGHAN) guidelines which established the position of EEN in pediatric CD (10, 19). A separate cluster around Arie Levine (20) and Rotem Sigall-Boneh (21) is also notable, which illustrates the ascendance of the CDED as a new therapeutic approach.

Citation burst detection provided a quantitative perspective on the shifting trends of research hotspots (Figure 11). The study of Arie Levine (20) also demonstrates the strongest burst (47.26) starting in 2020, indicating the paradigm-shifting effect of the CDED randomized controlled trial on clinical practice. Moreover, ongoing recent bursts (2021–2025) for Patrick F. van Rheenen (10), Dan Turner (22), and Henit Yanai (11) spotlight that the current emphasis in academia is very heavily focused on recent consensus guidelines and refining dietary management approaches for induction of remission.

## 4 Discussion

As far as we know, this study is the first comprehensive bibliometric analysis, which systematically shows the overall situation of global research on enteral and parenteral nutrition of IBD in the past 27 years. The number of papers published every year shows a power-law growth trend, especially after 2013. This trend reflects a major change in clinical concept: nutritional therapy has gone beyond the history as an auxiliary means to correct malnutrition and become a prominent focus of academic research and clinical exploration (23, 24). This change is closely related to the release of high-impact consensus guidelines, and it also benefits from people’s increasing recognition that dietary intervention is not only indispensable in inducing remission, but also essential for mucosal healing and improvement of long-term disease process (25, 26).

Geographically, academic research in this field is mainly concentrated in the North America and Europe, among which the USA and the United Kingdom are the core of international cooperation. Through spatial analysis, we found an obvious phenomenon: the number of papers published in China has increased rapidly, and now it ranks third in the world, but its TLS is much lower than that in western countries (China is 17, the United Kingdom is 140). While the USA leads in total publication volume, analyzing the average cooperation intensity (TLS per publication) reveals a different pattern. The United Kingdom exhibits the highest normalized cooperation index (0.55), significantly outperforming the USA (0.29) and China (0.10). This suggests that while China has achieved a “quantity” surge, its research remains relatively isolated compared to the highly collaborative networks seen in European nations. We acknowledge that raw publication counts are influenced by country size; however, even after normalizing for output volume, the disparity in international collaboration remains evident. Considering that IBD is becoming increasingly prevalent in newly industrialized countries, which is usually considered as the result of “westernization” of diet, this problem of insufficient cooperation is crucial (27–30). We must strengthen cross-continental cooperation to verify whether those western-centered dietary guidelines (such as ECCO/ESPGHAN) are completely suitable for Asians with different genes and eating habits (28, 29). Moreover, the central position and high density of institutions reflect their foundational contributions and sustained influence within the research network. Our keyword analysis highlights that pediatric Crohn’s disease and remission induction remain the primary focus areas of nutritional therapy research. This aligns with global guidelines prioritizing EEN to avoid steroid-related toxicity and promote growth in children.

Our co-occurrence analysis and sudden detection analysis show that the most striking change is the obvious change of treatment focus, from “bowel rest” to “microbiome modulation.” Early studies (1999–2010) mainly focused on TPN and corticosteroids, which reflected that nutrition was mainly regarded as a way to bypass the inflamed intestine at that time. After that, it entered the period of “EEN Revolution,” which can be seen from the continuous importance of Cluster # 0 (“exclusive enteral nutrition”) and the basic guidelines formulated by Frank M. Ruemmele et al., which established EEN as the gold standard for children’s mucosal healing (19). However, the most important recent development, marked by Arie Levine’s seminal and highly cited paper (intensity = 47.26), has turned to natural food-based therapies, such as the CDED (20, 31). This transformation has solved a key clinical problem faced by EEN-poor patient compliance caused by monotonous taste-and marked our step towards more sustainable and socially acceptable dietary interventions, which maintain high curative effect by excluding certain pro-inflammatory dietary ingredients (32, 33). From a clinical perspective, it is crucial to ground these bibliometric trends in real-world decision-making. For instance, the shift toward EEN and exclusion diets is visible in our maps, but it must be acknowledged that in adult practice, uptake remains heterogenous and is often limited by adherence, resources, and the need for multidisciplinary support. Furthermore, nutritional therapy does not evolve in isolation; it competes and interacts with biologics and small molecules. This should be framed alongside the broader therapeutic landscape, where newer agents such as JAK inhibitors are redefining treatment algorithms in refractory ulcerative colitis (34). Likewise, nutritional status is tightly linked to surgical risk, and perioperative outcomes in IBD are strongly influenced by malnutrition, steroids, and sepsis risk, connecting nutrition research with surgical prognostication and complication prevention (35).

Moreover, in the recent period (2020–2025), keywords such as “gut microbiome,” “fecal microbiota transplantation” and “modulation” have appeared, which shows that the research is deeper now. The current research is no longer satisfied with only seeing the patient’s symptoms improve; scholars pay more and more attention to the axis of “diet-microbiome-immunity” (36–39). This is related to the “quality of life” which attracts more and more attention, pointing to precision nutrition as the future of IBD care, characterized by strategies that strictly modulate the microbiome while allowing for personalization to sustain long-term dietary adherence (40, 41). This trajectory clearly indicates that next stage academic investigations will more heavily emphasize diet-based, personalized interventions and strategies for modulation with the microbiome.

There are several particularly powerful places in this research. It is the first attempt to systematically sort out the development of enteral and parenteral nutrition in patients with IBD in the past 27 years with comprehensive bibliometric methods and two databases (WoSCC and Scopus). Through the visual clustering technology of VOSviewer and CiteSpace, we have turned the complex literature data into an intuitive chart. In this way, the transition from “bowel rest” to “microbiome modulation” can be found accurately, and new hotspots such as Mediterranean diet and the CDED can be clearly seen, which provides a very useful direction reference for future research.

However, we should also know that it has some shortcomings. First of all, bibliometric analysis is mainly descriptive, which focuses on the number of papers and cooperation networks, but it is not deep enough in evaluating the methods of clinical evidence. Thus, it cannot replace meta-analyses to confirm evidence. In addition, regarding database coverage bias, our data collection is limited to the WoSCC/Scopus and English-published papers, which may miss some valuable non-English research or findings in regional databases, thus leading to selection bias. This study relied on bibliometric indicators (citation counts, TLS) without normalizing for country-specific economic or demographic factors (e.g., GDP, population size), which may influence the total research output of larger nations. Finally, our analysis is subject to inherent citation biases, such as self-citation, the Matthew effect, and journal prestige. Therefore, highly cited papers or bursts of research activity primarily reflect high academic attention rather than direct clinical efficacy.

Therefore, future research should focus on the following aspects: (1) Standardize the diet plan: formulate unified guidelines for new therapies such as CDED and Mediterranean diet, so that they can be used in more places around the world and are associated with higher patient adherence; (2) Understand the principle: deeply study how specific dietary ingredients affect the microorganisms and metabolites, thus helping the intestinal mucosa to heal; (3) Individualized therapy: predict people’s response to nutritional therapy by combining various omics data, and focus future research efforts toward precise nutrition; and (4) Enlarge the research population: conduct large-scale and multi-center RCTs specifically among adult patients and people of different races to verify whether nutritional therapy is also effective for people other than pediatric patients.

## 5 Conclusion

This paper systematically studies the papers on enteral and parenteral nutrition for IBD in the past 27 years, and shows the temporal evolution of core authors, subject clusters and research hotspots. The rapid increase in the number of papers shows that nutritional therapy has changed from being viewed merely as adjuvant therapy to becoming a major focus of academic and clinical research.

Furthermore, our bibliometric analysis highlights a significant evolution in research trends: a shift from EEN toward whole-food interventions, such as the CDED. This transition reflects an increasing academic emphasis on strategies that balance clinical efficacy with patient compliance. However, the current literature exhibits a pronounced skew toward pediatric CD. The lack of uniform dietary standards for adult patients, the scarcity of high-quality randomized controlled trials in non-Western populations, and the persistent research gap regarding Ulcerative Colitis remain significant challenges that must direct future academic endeavors.

## Data Availability

The raw data supporting the conclusions of this article will be made available by the authors, without undue reservation.
